# Maternal and Perinatal Factors Associated With Kawasaki Disease Among Offspring in Taiwan

**DOI:** 10.1001/jamanetworkopen.2021.3233

**Published:** 2021-03-26

**Authors:** Chaw-Liang Chang, Ming-Chih Lin, Ching-Heng Lin, Tai-Ming Ko

**Affiliations:** 1 Department of Biological Science and Technology, National Chiao Tung University, Hsinchu, Taiwan; 2 Department of Biological Science and Technology, National Yang Ming Chiao Tung University, Hsinchu, Taiwan; 3 Department of Pediatrics, Cathay General Hospital, Hsinchu, Taiwan; 4 School of Medicine, Fu-Jen Catholic University, New Taipei City, Taiwan; 5 Department of Pediatrics, Taichung Veterans General Hospital, Taichung, Taiwan; 6 School of Medicine, National Yang Ming Chiao Tung University, Taipei, Taiwan; 7 Department of Food and Nutrition, Providence University, Taichung, Taiwan; 8 School of Medicine, Chung Shan Medical University, Taichung, Taiwan; 9 Department of Medical Research, Taichung Veterans General Hospital, Taichung, Taiwan; 10 Department of Industrial Engineering and Enterprise Information, Tunghai University, Taichung, Taiwan; 11 Department of Health Care Management, National Taipei University of Nursing and Health Sciences, Taipei, Taiwan; 12 Department of Public Health, College of Medicine, Fu Jen Catholic University, New Taipei City, Taiwan; 13 Institute of Public Health and Community Medicine Research Center, National Yang Ming Chiao Tung University, Taipei, Taiwan; 14 Institute of Bioinformatics and Systems Biology, National Chiao Tung University, Hsinchu, Taiwan; 15 Institute of Biomedical Sciences, Academia Sinica, Taipei, Taiwan; 16 Graduate Institute of Integrated Medicine, College of Chinese Medicine, China Medical University, Taichung, Taiwan; 17 Center For Intelligent Drug Systems and Smart Bio-devices, National Yang Ming Chiao Tung University, Hsinchu, Taiwan; 18 Drug Development and Value Creation Research Center, Center for Cancer Research, Kaohsiung Medical University, Kaohsiung, Taiwan

## Abstract

This case-control study investigates the association of perinatal factors and maternal autoimmune diseases with the development of Kawasaki disease using the Taiwan Maternal and Child Health Database.

## Introduction

Kawasaki disease (KD) is the most recognized childhood vasculitis and the leading cause of pediatric-acquired heart disease in developed countries.^1^ Increased KD incidence among East Asian children, a high risk among siblings and twins, and familial occurrence suggest a genetic predisposition.^1,2^ However, genetic factors alone cannot explain seasonal variations, periodic outbreaks, or the continued increase in KD incidence.^2^ Many other factors, including exposure to infectious agents, pollution, and elevated atmospheric biological particle concentrations, are associated with KD.^2^

Several studies^3,4^ have suggested that maternal and perinatal factors might be associated with KD development. This case-control study investigated the role of perinatal factors and maternal autoimmune diseases in the development of KD using the Taiwan Maternal and Child Health Database.

## Methods

The institutional review board of the Cathay General Hospital approved this study and waived the need for informed consent because the data are publicly available and deidentified. The study followed the Strengthening the Reporting of Observational Studies in Epidemiology (STROBE) reporting guideline for case-control studies.

We collected data of patients who were younger than 5 years, had KD, and were born between 2004 and 2010, from the original claims data of the Taiwan National Health Insurance Research Database, which was established in 1995 and covered 99.9% of the population by 2003.^5^ KD diagnosis (*International Classification of Diseases, Ninth Revision* code 446.1) was confirmed by receipt of intravenous immunoglobulins (Anatomic Therapeutic Chemical code J06BA02) and hospitalization. Age-matched and index date–matched individuals were collected as a control group. Every citizen in Taiwan is assigned a unique national identification number. The Taiwan Maternal and Child Health Database, which contains national identification numbers of children and their parents, was used to link parents and children.^5^ The child’s birth history, maternal comorbidities, and maternal autoimmune diseases were analyzed (see the eAppendix in the Supplement).

Differences in categorical and continuous variables were assessed using χ^2^ and *t* tests, respectively. *P* values were 2-sided, and statistical significance was set at *P *<* *.05. Analyses were performed using SAS Enterprise Guide statistical software version 9.4 (SAS Institute). The analyses were conducted from May 1 to August 6, 2020.

## Results

We enrolled 4197 patients with KD (2601 boys [62.0%]; 1717 [40.9%] younger than 1 year; 1261 [30.0%] aged 1 year) and 16 788 matched individuals without KD (8832 boys [52.6%]) from 1 280 374 children in the database. Male sex (odds ratio [OR], 1.47; 95% CI, 1.37-1.57; *P* < .001), maternal age 35 years or older (OR, 1.18; 95% CI, 1.07-1.30; *P* < .001), maternal Sjögren syndrome (OR, 1.75; 95% CI, 1.03-2.95; *P* = .04), and maternal ankylosing spondylitis (OR, 2.01; 95% CI, 1.17-3.43; *P* = .01) were associated with increased KD risk in the offspring. However, low birth weight, preterm delivery, other maternal autoimmune diseases, and maternal comorbidities showed no associations with risk of developing KD (Table 1). In the multivariable analysis, maternal ankylosing spondylitis was associated with a 2.02 times higher odds of KD in the offspring (95% CI, 1.18-3.47; *P* = .01) (Table 2).

**Table 1.** Characteristics of Study Participants and Factors Associated With KD

Characteristic	Participants, No. (%)		OR (95% CI)	*P* value
	Non-KD group (n = 16 788)	KD group (n = 4197)		
Age, y				
0	6868 (40.9)	1717 (40.9)	NA^a^	NA^a^
1	5044 (30.0)	1261 (30.0)		
2	2336 (13.9)	584 (13.9)		
3	1180 (7.0)	295 (7.0)		
4	824 (4.9)	206 (4.9)		
5	536 (3.2)	134 (3.2)		
Sex				
Female	7956 (47.4)	1596 (38.0)	1 [Reference]	<.001
Male	8832 (52.6)	2601 (62.0)	1.47 (1.37-1.57)	
Birth weight, g				
≥2500	15 672 (93.4)	3929 (93.6)	1 [Reference]	.55
<2500	1116 (6.6)	268 (6.4)	0.96 (0.84-1.10)	
Mother’s age, y				
<35	14 611 (87.0)	3571 (85.1)	1 [Reference]	<.001
≥35	2177 (13.0)	626 (14.9)	1.18 (1.07-1.30)	
Preterm delivery, wk				
≥37	15 448 (92.0)	3856 (91.9)	1 [Reference]	.76
<37	1340 (8.0)	341 (8.1)	1.02 (0.90-1.15)	
Maternal comorbidity				
Diabetes	125 (0.7)	27 (0.6)	0.86 (0.57-1.31)	.49
Hypertension	143 (0.9)	39 (0.9)	1.09 (0.77-1.56)	.63
Hyperlipidemia	202 (1.2)	54 (1.3)	1.07 (0.79-1.45)	.65
Gestational diabetes	272 (1.6)	60 (1.4)	0.88 (0.67-1.17)	.38
Gestational hypertension	84 (0.5)	23 (0.5)	1.10 (0.69-1.74)	.70
Preeclampsia or eclampsia	188 (1.1)	51 (1.2)	1.09 (0.80-1.48)	.60
Maternal autoimmune disease				
Systemic lupus erythematosus	47 (0.3)	10 (0.2)	0.85 (0.43-1.69)	.64
Rheumatoid arthritis	55 (0.3)	11 (0.3)	0.80 (0.42-1.53)	.50
Sjögren syndrome	46 (0.3)	20 (0.5)	1.75 (1.03-2.95)	.04
Ankylosing spondylitis	40 (0.2)	20 (0.5)	2.01 (1.17-3.43)	.01
Psoriatic arthritis or psoriasis	43 (0.3)	9 (0.2)	0.84 (0.41-1.72)	.63
Autoimmune thyroiditis	26 (0.2)	9 (0.2)	1.39 (0.65-2.96)	.40

Abbreviations: KD, Kawasaki Disease; NA, not applicable; OR, odds ratio.

^a^ The groups were age matched so no comparisons were made.

**Table 2.** Multivariable Analysis of Autoimmune Diseases Associated With Kawasaki Disease

Autoimmune disease	Model 1^a^		Model 2^b^	
	OR (95% CI)	*P *value	OR (95% CI)	*P* value
Systemic lupus erythematosus	0.85 (0.43-1.67)	.63	0.86 (0.43-1.71)	.67
Rheumatoid arthritis	0.78 (0.41-1.49)	.45	0.79 (0.41-1.52)	.49
Sjögren syndrome	1.74 (1.03-2.94)	.04	1.67 (0.99-2.84)	.06
Ankylosing spondylitis	1.97 (1.15-3.37)	.01	2.02 (1.18-3.47)	.01
Psoriatic arthritis or psoriasis	0.85 (0.41-1.74)	.65	0.85 (0.41-1.74)	.65
Autoimmune thyroiditis	1.38 (0.65-2.94)	.41	1.40 (0.65-2.99)	.39

Abbreviation: OR, odds ratio.

^a^ Model 1 was adjusted for neonatal sex and maternal age.

^b^ Model 2 was adjusted for neonatal sex, maternal age, neonatal age, birth weight, preterm delivery, and maternal comorbidity.

## Discussion

Given that the age of KD onset is between 6 months and 5 years, and it is most severe during the first year of life, it is possible that the child’s immature immune system and maternal and perinatal factors might be associated with KD development. However, the identification of maternal associations, especially when investigating perinatal factors associated with pediatric diseases, is the challenge when integrating different large databases to link maternal health information with the child’s clinical phenotypes.^5^ In this study, we found that advanced maternal age was significantly associated with KD development in the offspring. This association may partly explain the increasing KD incidence in developed countries because ages at marriage and childbearing are increasing. The advanced parental age may be associated with more germline de novo variants, which may lead to KD in the offspring.^6^ Furthermore, we demonstrated that maternal ankylosing spondylitis and Sjögren syndrome may be perinatal factors associated with increased risk of KD. This suggests that a maternal autoimmune disease or its associated medical treatment might induce an epigenetic predisposition to developing KD in the offspring.

The main limitation of the study was the unavailability of the genetic and environmental confounders.^1,2^ Moreover, our findings are based on data of patients who were younger than 5 years. Therefore, the role of maternal factors in increasing the risk of KD in offspring requires further investigation, especially in older patients.

## Notes

Supplement. **eAppendix. **Supplemental Methods
