# Supplementary material for: Maternal and Perinatal Factors Associated With Kawasaki Disease Among Offspring in Taiwan
Source: JAMA Netw Open. 2021 Mar 26;4(3):e213233. doi: 10.1001/jamanetworkopen.2021.3233 (PMC7998075; doi:10.1001/jamanetworkopen.2021.3233)
Supplement: Supplement. — eAppendix. Supplemental Methods [file jamanetwopen-e213233-s001.pdf]

Supplemental Online Content

Chang CL, Lin MC, Lin CH, Ko TM. Maternal and perinatal factors associated with Kawasaki disease among offspring in Taiwan. *JAMA Netw Open*. 2021;4(3):e213233. doi:10.1001/jamanetworkopen.2021.3233

eAppendix. Supplemental Methods

This supplemental material has been provided by the authors to give readers additional information about their work.

© 2021 Chang CL et al. *JAMA Network Open.*

eAppendix. Supplemental Methods

Data on maternal diseases before delivery were collected, including diabetes mellitus, hypertension, hyperlipidemia, gestational diabetes mellitus, gestational hypertension, pre-eclampsia or eclampsia, systemic lupus erythematosus, rheumatoid arthritis, Sjögren's syndrome, ankylosing spondylitis, psoriatic arthritis/psoriasis and autoimmune thyroiditis. We matched 4 controls per case in this study to maximize the power of precise effect estimation. A multivariable logistic regression model was used to investigate the odds ratio (OR). We calculated the odds ratios and 95% confidence intervals to assess whether the examined factors were statistically different between individuals with and without Kawasaki disease (KD). In model 1, we adjusted the OR for neonatal sex and maternal age because these two factors increased KD risk in the offspring. In model 2, we adjusted the OR for all factors, including maternal age, neonatal age, neonatal sex, birth weight, preterm delivery, and maternal comorbidity. Analyses were performed using SAS Enterprise Guide statistical software version 9.4 (SAS Institute).

© 2021 Chang CL et al. *JAMA Network Open.*
